# Thermally Promoted Cation Exchange at the Solid State in the Transmission Electron Microscope: How It Actually Works

**DOI:** 10.1021/acsnano.3c04516

**Published:** 2023-08-28

**Authors:** Alberto Casu, Miquel Lopez, Claudio Melis, Davide Deiana, Hongbo Li, Luciano Colombo, Andrea Falqui

**Affiliations:** ‡ Department of Physics “Aldo Pontremoli”, University of Milan, Via Celoria 16, 20133 Milan, Italy; † Biological and Environmental Sciences and Engineering (BESE) Division, King Abdullah University of Science and Technology (KAUST), Nabla Lab, Thuwal 23955-6900, Saudi Arabia; § Department of Physics, University of Cagliari, Cittadella, University of Cagliari, Cittadella Universitaria di Monserrato, 09042 Monserrato (CA), Italy; # Centre Interdisciplinaire de Microscopie Électronique (CIME), Ecole Polytechnique Fédérale de Lausanne (EPFL), 1015 Lausanne, Switzerland; ∥ Experimental Center of Advanced Materials, School of Materials Science and Engineering, Beijing Institute of Technology, Beijing 100081, China; ⊥ Interdisciplinary Centre for Nanostructured Materials and Interfaces (CIMaINa), Department of Physics “Aldo Pontremoli”, University of Milan, Via Celoria 16, 20133 Milan, Italy

**Keywords:** cation exchange, nanocrystals, solid state reactions, in situ heating scanning transmission electron microscopy, EDS and EELS chemical mapping, molecular dynamics simulations

## Abstract

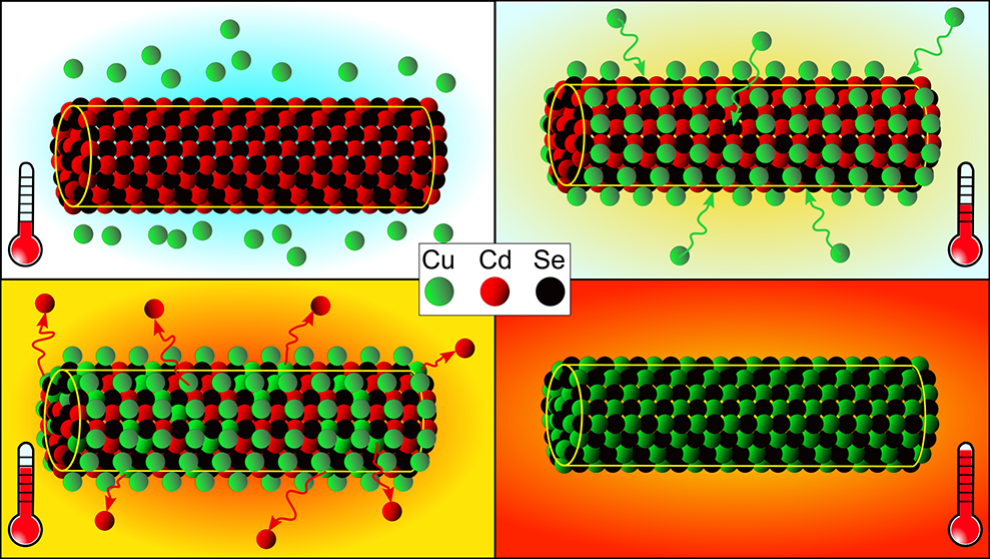

Cation exchange offers a strong postsynthetic tool for nanoparticles that are unachievable via direct synthesis, but its velocity makes observing the onset of the reaction in the liquid state almost impossible. After successfully proving that cation exchange reactions can be triggered, performed, and followed live at the solid state by an *in situ* transmission electron microscopy approach, we studied the deep mechanisms ruling the onset of cation exchange reactions, i.e., the adsorption, penetration, and diffusion of cations in the host matrices of two crystal phases of CdSe. Exploiting an *in situ* scanning transmission electron microscopy approach with a latest generation heating holder, we were able to trigger, freeze, and image the initial stages of cation exchange with much higher detail. Also, we found a connection between the crystal structure of CdSe, the starting temperature, and the route of the cation exchange reaction. All the experimental results were further reviewed by molecular dynamics simulations of the whole cation exchange reaction divided in subsequent steps. The simulations highlighted how the cation exchange mechanism and the activation energies change with the host crystal structures. Furthermore, the simulative results strongly corroborated the activation temperatures and the cation exchange rates obtained experimentally, providing a deeper understanding of its phenomenology and mechanism at the atomic scale.

In the last two decades, the need for innovative nanomaterials has dramatically increased for their versatility in a wide range of applications such as optoelectronics, photonics, and catalysis.^1−6^ Therefore, several robust and effective synthetic methods have been developed for obtaining nanocrystals (NCs) with very finely tuned compositions, crystal phases, and morphologies.^7−10^ Among the diverse approaches that have matured in recent years, some consist of postsynthetic transformations of preformed nanostructures, thus allowing one to obtain nanomaterials with features not directly attainable by *de novo* synthetic methods.^11−13^ Among these postsynthetic modifications, cation exchange (CE) reactions permit the substitution of cations within a host NC lattice with those in solution, and these have proven to be a potent tool to obtain very fine control over NC compositions and phases.^14−17^ Revealing the underlying mechanism is of great importance to guide the rational design of nanostructures in a precise way. Previously, several indirect yet feasible ways have been proposed by analyzing the intermediate sample generated during the CE reaction in liquid states. However, these approaches are inherently limited by two factors, namely, the velocity of the CE reaction performed in liquid and the limited capability of sampling its intermediate states by extracting aliquots while it is occurring. For these reasons, observations of CE reactions at the solid state with an *in situ* transmission electron microscopy (TEM) approach provide a more reliable way to continuously monitor the CE reaction with a highly improved level of detail over a time scale that is not accessible in classical conditions, due to the slower kinetics of the solid state CE reaction and the fast sampling granted by TEM. In particular, in our past work^18^ we first showed that CE reactions can be performed at the solid state, by triggering and monitoring their evolution with a TEM-based *in situ* heating strategy that allowed unique control over its intermediate states. Here, two distinctly shaped populations of nanoparticles were separately drop-cast on a TEM grid, one composed of Cu_2_Se or Cu spherical NCs with a face-centered cubic zincblende (zb) crystal structure acting as a cation donor and CdSe nanorods (NRs) or nanowires (NWs) with a hexagonal wurtzite (wz) structure as the cation acceptor. The difference in shape and size between the two populations helped make them immediately recognizable, while drop-casting each population at a different time determined a random distribution of donor and acceptor nanoparticles across the film. Once we heated a standard TEM grid to *T* = 400 °C with an oven-based *in situ* heating specimen holder, which acted as a substrate for both Cu_2_Se NCs and CdSe NWs, we observed that the Cu_2_Se/Cu NCs partially expelled copper. The expelled copper diffused on the grid and entered the wz CdSe NRs/NWs, giving rise to CE reactions in the solid state, i.e., replacing the Cd and transforming the wz CdSe into zb Cu_2_Se.

In the present work, we expand the scope of those first *in situ* heating studies^18^ to gain a deeper understanding of the general mechanism of the CE reaction and of the early stages that correspond to the onset of CE. We face this challenge by moving along different paths:

(i) By performing further CE *in situ* experiments at the solid state with a modern, microelectronic mechanical system (MEMS)-based *in situ* heating holder, capable of fast heating and cooling of the sample with almost zero spatial drift. This allowed us to record the early stages and possibly the onset of CE, which could not be observed during our previous experiments due to the technical limitations of a classic oven-based TEM holder.^16^

(ii) By adopting a (high resolution) scanning TEM (HR)STEM-based imaging strategy, where the local chemical composition was probed with much higher spatial resolution by electron energy loss (EELS) and energy dispersive X-ray (EDS) spectroscopy maps.

(iii) By introducing zb CdSe NWs paired with Cu_2_Se NCs, in order to verify how a different starting phase changed the role of the acceptor crystal structure with regards to the CE reaction.

(iv) By explaining several and peculiar phenomena experimentally observed by an in-depth theoretical study, aimed at atomistically modeling the whole process of CE reaction at the solid state by classical molecular dynamics (MD).

We found several unexpected results. First, both HRSTEM-EELS/EDS chemical mappings showed that the copper expelled by the Cu_2_Se NCs forms a thin layer around the CdSe NWs after migrating over the heated substrate and before entering the NWs, even when the temperature is still quite low. Indeed, we reported in our previous paper that the expulsion of copper became detectable at 400 °C.^18^ Moreover, since the CE reaction replacing Cd with Cu in the wz CdSe started at the same temperature, we did not determine the actual thermal onset corresponding to the Cu_2_Se NPs making copper available for entering the CdSe NWs and replacing Cd. However, as in the case of zb CdSe NWs, we found that the same CE reaction can start below 125 °C; this also implies that some copper has been already expelled by the Cu_2_Se NPs at that temperature. Besides, upon further heating, the copper enters the CdSe NWs as expected. However, major differences emerge in the CE reaction, depending on the crystal structure of the CdSe constituting the NWs. Indeed, while the CE reaction was confirmed to happen at 400 °C for the CdSe with wz structure, the diffusion with concomitant Cd replacement occurs at 125 °C in the zb CdSe NWs. Moreover, in the NWs constituted by zb CdSe, the substitution of Cd ions with Cu was observed to be partially reversible in the low temperature regime, i.e., below 250 °C. In other words, we found that, up to that temperature, the smaller domains of Cu_2_Se can be transformed back into CdSe, while the bigger ones keep growing in length. Aiming at better understanding these results, the whole CE reaction was simulated by a classical molecular dynamics (MD) approach, splitting it into different steps, corresponding to the diverse and succeeding phenomena we experimentally observed. The MD simulations provided numerical estimations of the activation energies for each reaction step. In turn, this allows one (i) to calculate the probabilities of the whole CE reaction for the cubic and hexagonal crystal phases of the CdSe NWs and (ii) to clarify how these probabilities correspond to diverse thermal-dependent rates of the CE reaction. Furthermore, the present simulations provided physical insights on the partial reversibility of the CE reaction observed in the smaller Cu_2_Se domains when the temperature was kept below 250 °C. The twofold experimental and theoretical approach we followed in this work provides a solid framework for both imaging with much higher detail the CE at the solid state and interpreting it as the overall result of several physical processes. In particular, the disassembly of CE in subsequent steps clarifies the factors at play in determining the activation temperatures for CE reactions with respect to the host crystal structures in different conditions.

## Results and Discussion

### Improvement and Aims of the *In Situ* Experimental Approach

The choice of adopting MEMS heating chips instead of mounting TEM grids in an oven-based heating holder allowed a different approach toward *in situ* heating experiments, based on the possibility of very precise control over the holder temperature. This implies the capability of fast halting of the heating ramp to a steady temperature of choice or fast cooling it to stop and freeze the temperature-triggered reaction without relevant thermal latency and with almost zero spatial drift.

The different starting points and goals of two parallel studies, the first one using wz CdSe NWs and the second one replacing them with zb CdSe ones, also lead to differentiating the experimental techniques adopted in each case. Then, since the overall evolution of CE where wz CdSe NWs transformed into zb Cu_2_Se has already been studied in general terms and over wide areas,^18^ we devoted our attention toward studying its early stages over smaller areas by STEM-EELS-based spectrum imaging. On the other hand, we approached the system where cubic zb CdSe NWs were transformed into Cu_2_Se, by combining HRSTEM and STEM-EDS mapping to follow the evolution of CE from both a structural and compositional point of view. Since the main aim of chemical analysis was irrespective of the spectrometry used to obtain the elemental maps, we decided to move from STEM-EELS to STEM-EDS chemical mapping for technical reasons. This choice was dictated by the effectiveness of the drift correction, which in our microscope was more performing in the STEM-EDS mapping than in the STEM-EELS mapping, and took into account the composition of the MEMS chips used to heat the mixture of NCs containing zb CdSe NWs (see Methods section for further information), improving the resolution of the chemical maps while maintaining the capability of observing the early stages of CE.

Finally, considering that our main interest lies in the early stages of the CE reactions to pinpoint the mechanisms that rule over the arrival of the Cu species on and in the CdSe NWs, all the *in situ* experiments were conducted on relatively Cu-poor systems, i.e., populations with lower concentrations of donor Cu_2_Se nanospheres and similar amounts of acceptor wz or zb CdSe NWs, respectively. In this regard, by using metal Cu NPs instead of the Cu_2_Se ones, in our previous work,^18^ we showed that lowering the amount of copper available for CE did not impact the occurrence of the reaction and its outcome. Thus, in this work, we lowered the amount of available copper only to slow down the reaction rate of CE, so that we could investigate it in greater detail.

### CE between Cu_2_Se and Wurtzite CdSe NWs

CE at the solid state was previously observed *in situ* for the Cu_2_Se and wz CdSe system^18^ as a temperature-activated process characterized by a sharp thermal threshold, so that the whole process takes place between 350 and 400 °C if we consider the temperature interval between its earliest onset and its completion.

**Figure 1:**
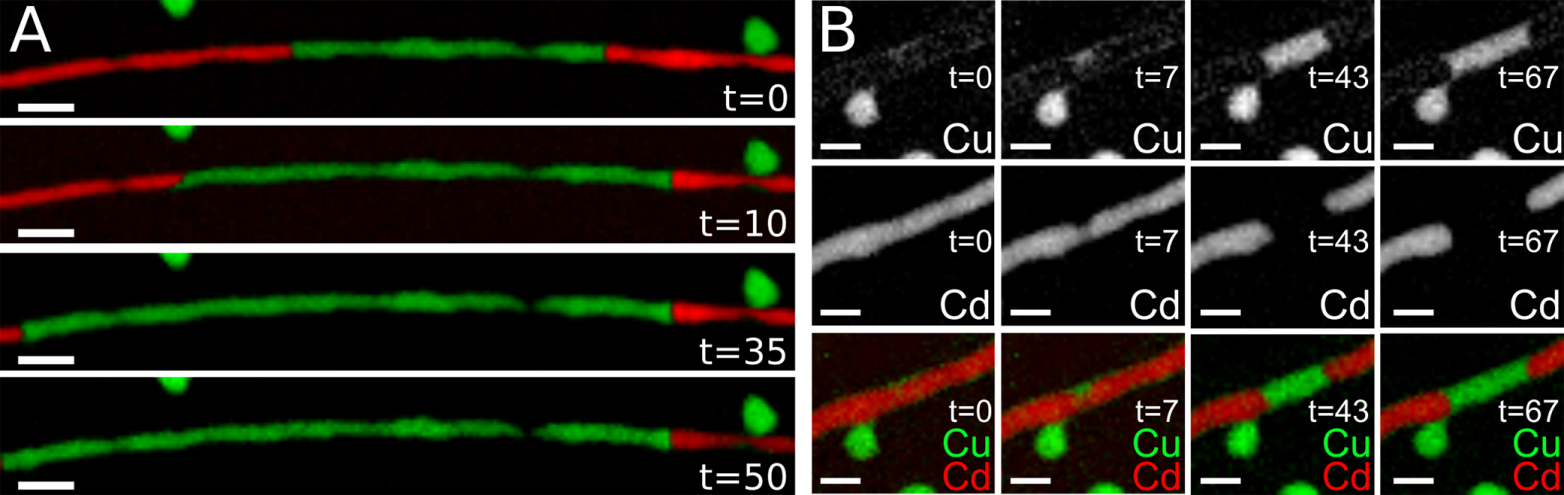
Cd–Cu CE evolution in wz NWs over time at constant *T* = 400 °C after a blind CE experiment (A) and with a decreased amount of available Cu (B). STEM-EELS maps of Cu and Cd are shown at diverse times from the reaction beginning, where the time is indicated in minutes. Scale bars are 50 nm (A) and 10 nm (B).

The first step was checking a square-wave approach to heating ramps for CE reactions between Cu_2_Se and wz CdSe by depositing both populations on a heating MEMS chip and driving it to the final temperature, previously identified by classical oven-based heating. Thus, a blind CE experiment was performed by quickly approaching the final temperature of 400 °C and keeping it steady for 5 min with the beam blanked. The temperature was then brought back to RT to freeze the CE reaction and look by STEM-EELS mapping for a partially exchanged CdSe NW without Cu_2_Se NCs adjacent to the exchanged zone (Figure 1A). Next, the temperature was quickly raised back to 400 °C and the local evolution of the CE reaction was followed over time. As the CE reaction evolved over time, it became apparent that the Cu_2_Se nanospheres do not play a direct role in the CE reaction. In fact, no entry point for CE ever forms in the proximity of the Cu_2_Se NC at the right end of the field of view and the CE front (i.e., where Cu is substituting Cd) does not move faster on the right-hand side of the NW due to the Cu_2_Se nanosphere’s presence.

*In situ* heating CE reaction experiments were repeated by decreasing the amount of spherical Cu_2_Se NCs (and thus Cu available for the CE reaction) to study the onset and the early evolution of the CE reaction with greater clarity, as shown in Figure 1. Again, the final temperature of 400 °C was quickly reached to boost the onset of CE and chemical maps of Cu and Cd were recorded. A faint Cu signal can be observed around the CdSe NW even before the actual CE starts, and this “shadow” covering becomes increasingly better-defined over time and is consistent with a shell-like, superficial distribution (Figure SI_1). A comparable trend can also be observed by looking at the entry point for the CE in the NW: at first a slight, thorn-like copper signal can be observed inside the NW, which becomes clearly visible before making way for a NW-wide CE front that moves along the length of the NW. Splitting the combined elemental maps to single elements highlights the evolution of the copper signal, making it apparent that the entry point for copper is not related to the Cu_2_Se NC in proximity to the NW, but must be related to an intrinsic local state of the NW. Moreover, by looking at the Cd maps, it must be noted that the Cd signal in the NW becomes locally weaker and disappears in correspondence with the regions where Cu appears, indicating once again that a mixed Cu/Cd state can be eventually observed only at the CE front but is not maintained after the CE front has passed (Figure 1B).

### CE between Cu_2_Se and Zincblende CdSe Wires

Following the results of the study involving wz CdSe as a cation acceptor, further experiments were developed by keeping Cu_2_Se nanospheres as cation donors and modifying the phase of the NW acceptor species to cubic zb CdSe (Figure SI_2), with the goal of verifying if CdSe still undergoes a CE reaction at the solid state and under which conditions. First off, this allowed verifying how free copper acts under heating in similar environmental conditions and in the presence of an acceptor population with a structure that more closely resembles that of Cu_2_Se. Besides, these experiments aimed to confirm that free copper moving on the film toward the CdSe NWs for the CE reaction still forms a thin layer around them before the actual CE reaction starts, even in the presence of a different phase of CdSe, since it had already been proven that the reaction was not influenced by different substrates.^18^

**Figure 2:**
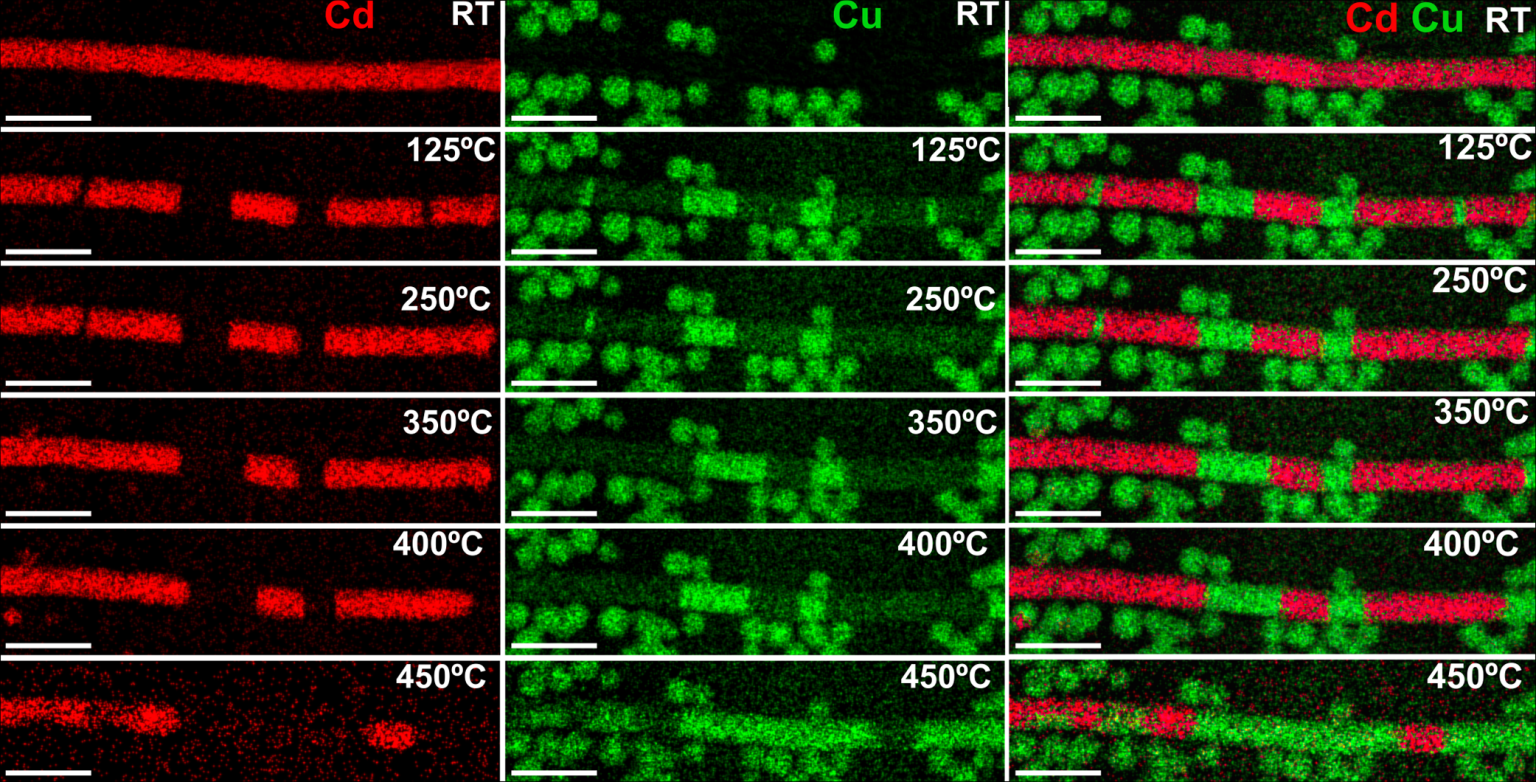
STEM-EDS elemental maps of representative CdSe NWs and Cu_2_Se nanospheres during *in situ* heating at an increasing temperature. Single element maps of Cd (red) and Cu (green) are reported in the left and center columns, and a combined Cd+Cu map is reported in the right-side column. Scale bars are 50 nm.

**Figure 3:**
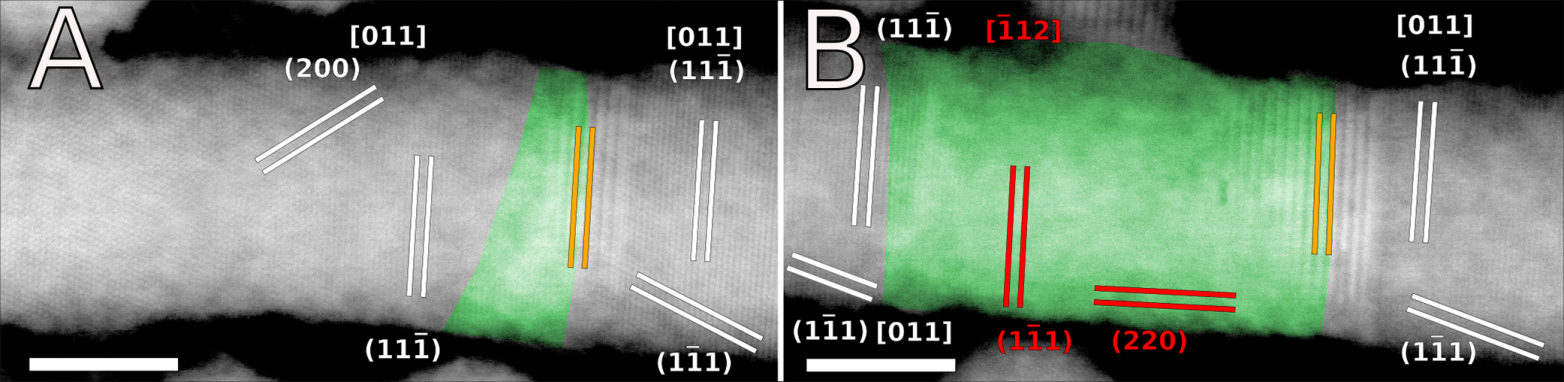
HRSTEM image of the small, thorn-shaped CE front (A) and of the big CE front (B) already observed by STEM-EDS elemental maps at 125 °C, where some lattice planes of Cu_2_Se (red) and cubic CdSe (white) are also reported. The green zones correspond to the part of the NW where Cu replaced Cd. Scale bars are 10 nm.

As displayed in Figure 3, upon heating the sample to 125 °C, STEM-EDS maps show that multiple CE exchange fronts appear in the NW. By further heating the system, it could be observed that the progression of the different CE fronts and the CE reaction is not limited to a straightforward advancement along the length of the NW. In fact, the position and extension of the exchanged domains of the NW vary over time and temperature, with the smaller Cu_2_Se domains reverting back to CdSe and the bigger ones slowly growing with the increasing temperature until finally merging at 450 °C. This experimental evidence is even clearer in the single element maps, which also show that the Cu covering is once again present along the length of the wire. Intensity profiles recorded perpendicularly to the length of the NW once again show the typical M-shape that should be expected in the presence of a superficial, shell-like distribution of Cu (Figure SI_1). This sort of additional shell is already clearly visible at 125 °C in the map of the sole copper, and it is maintained throughout the whole heating ramp, almost disappearing from view only when shadowed by the exchanged Cu-rich regions. Given the wavering composition of the smallest domains undergoing CE in the intermediate stages, which flip between Cu_2_Se and CdSe, this experimental evidence indicates that the external Cu covering is maintained, even when the exchanged regions revert back from Cu_2_Se to CdSe (Figure 2).

Furthermore, by splitting the elemental maps into two sets of single element maps, it is possible to clearly observe that once again the CE fronts apparently begin from local points in the NW acting as preferential entries for Cu (see, for example, the side entry points on the left side of the Cu map recorded at 400 °C in Figure 2). The thorn-like CE front then expands to cover the whole width of the NW and form proper plane CE fronts across the NW. Also, these plane CE fronts either disappear if their size is small or move along the NW’s length, pushing forward the CE while keeping plane interfaces between the exchanged and nonexchanged portions of the NW.

In agreement with the results previously obtained for wz CdSe, the structural evolution of the zb CdSe NWs was also studied by HRSTEM to assess which phases can be observed once the CE manifests. The corresponding imaging data are reported in Figure 3. The two-dimensional fast Fourier transform (2D-FFT) analysis of relevant regions of interest (ROI) (not shown here) allowed one to appreciate local variation in the interplanar distances and relative orientations occurring between different lattice sets in the original CdSe NW, in its exchanged portions and in the Cu_2_Se nanospheres. More in detail, the HRSTEM data were recorded at RT after the temperature had already been set at 125 °C, thus freezing the reaction at its early stages during the acquisition of HRSTEM imaging. In particular, the beginning of the CE reaction was also studied from a structural point of view on the small, thorn-like CE front (Figure 3A) and on the major CE front (Figure 3B) observed on the left side and in the middle of the NW reported in the elemental maps in Figure 2, respectively. The cation exchanged zones previously observed by STEM-EDS mapping are depicted in false color as green.

Comparative structural analysis of both zones shows that the portions of the NW corresponding to the CE fronts display a striking local variation in the lattice spacing, which manifests as wider dark/lights fringes that seem to maintain the same orientation observed for the (111̅) atomic planes of zb CdSe in the nonexchanged zones indicated as orange lines in Figure 3. This occurrence can be explained as the manifestation of Moiré patterns, which are caused by the interference of two sets of lines with nearly common periodicity and limited angular mismatch.

Then, the wide lines observed in these zones (7.40 Å) can be effectively identified as Moiré fringes caused by the superposition of the (111̅) planes of cubic Cdse and Cu_2_Se (*d*_CdSe_= 3.51 Å and *d*_Cu2Se_ = 3.37 Å, respectively) if the general formula
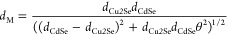
1is satisfied. Here *d*_M_ indicates the supposed Moiré fringes (*d*_M_ = 7.40 Å), *d*_CdSe_ and *d*_Cu2Se_ indicate the (111̅) atomic planes of CdSe and Cu_2_se, respectively, and θ indicates the angular mismatch between the two sets of atomic planes.

The mandatory condition indicated by eq 1 is satisfied by using the aforementioned values of *d*_M_, *d*_CdSe_ and *d*_Cu2Se_ (the latter of which are also consistent with values reported in the diffraction cards of CdSe (JCPDS card no. 19-0191) and Cu_2_Se (JCPDS card no. 79-1841)), and the resulting angular mismatch between CdSe and Cu_2_Se planes can be calculated as θ ≅ 1.2°. Then, the presence of Moiré fringes around the compositional interfaces indicated by the elemental maps in Figure 3, irrespective of the size of the exchanged domains, indicates that the CE reaction determines a minor rearrangement in the crystal lattice at its front, which gets reabsorbed once the exchanged Cu settles in the NW to form ordered Cu_2_Se domains. Moreover, since HR(S)TEM images are bidimensional projections of tridimensional NWs, the Moiré fringes suggest that the mild structural perturbations associated with CE do not manifest as planar fronts but likely give rise to exchanged wedges into the nonexchanged zones.

Some considerations must be taken into account about the possible differences between the above-described steps we found constituting the investigated CE reaction at the solid state and the mechanism it should follow when performed in a liquid solution. As previously highlighted, monitoring and understanding a CE reaction involving NCs in solution is very hard. In fact, due to its short time, capturing intermediate steps and obtaining reliable data would be very difficult when CE is performed in a liquid environment. To overcome this limitation, we adopted solid state thermally promoted *in situ* CE. Thus, one could wonder if when performed in liquid the same CE reaction follows an analogous path. With this regard, even with the possibility of performing *in situ* liquid STEM imaging of that reaction, its short time still would pose a constraint, since acquiring acceptable elemental maps takes many seconds to minutes, resulting in monitoring with a high electron dose a continuously changing situation, which in the liquid environment could not be easily stopped. In addition to time constraints, factors like the resolution decrease, due to the electron scattering with the whole liquid thickness, and Brownian motion of NWs, due to the solvent temperature, further complicate *in situ* liquid STEM imaging.^19^ Furthermore, possible electron-beam-induced NCs modifications, expected electron dose-dependent radiolysis of the liquid environment (i.e., solvent and compounds it contains), and possible heating effects are expected to concomitantly change how the CE reaction occurs in an unpredictable way.^19^ Ultimately, the authors chose to study it in vacuum and in the solid state, since currently this appears to be the only way to attain both high spatial and temporal resolution.

### Atomistic Modeling of Cation Exchange by Molecular Dynamics

The *in situ* experiments showed that the overall mechanism of CE consisted of a sequence of concerted phenomena: (i) the absorption of a Cu shell on the CdSe NWs after Cu atom expulsion from the Cu_2_Se NCs, (ii) the time evolution of the Cu front within the NW, and (iii) the actual CE mechanism, for which the rate depends on the underlying CdSe crystal structure and temperature.

**Figure 4:**
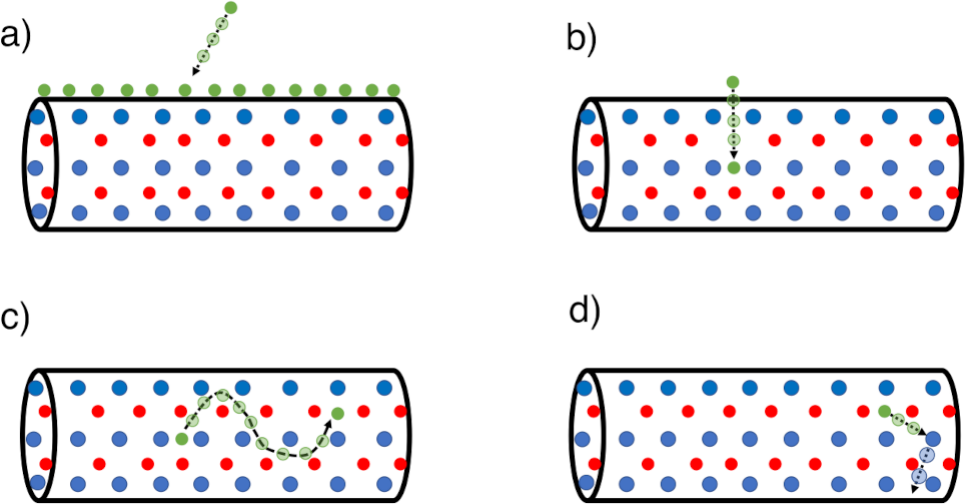
Schematic of the adopted model for the whole CE process based on four steps: (a) formation of a thin Cu shell around a CdSe NW; (b) penetration of Cu atoms into the CdSe NW; (c) diffusion of a Cu interstitial defect within the CdSe matrix; (d) Cu → Cd substitution via a kick-off event.

In order to provide a rationale for such phenomena and to work out a general theoretical picture for them, we developed an atomistic model based on classical MD simulations aimed at (i) identifying specific local mechanisms involved in the CE process and (ii) quantifying their specific contribution to the whole process. Thus, starting from the phenomenology observed in both past and current *in situ* experiments, we modeled the whole CE reaction as four succeeding steps (see Figure 4): (a) the formation of a Cu shell around a CdSe NW; (b) the penetration of Cu atoms into the CdSe NW, and the subsequent creation of Cu interstitial defects; (c) the diffusion of Cu interstitial defects within the CdSe matrix; (d) the final Cu → Cd replacement via a kick-off event. Each of these specific steps was simulated, thus allowing an estimation of the corresponding activation energies.

The interatomic interactions in CdSe and Cu_2_Se have been modeled by a combination of a Lennard-Jones and a Coulombic term. The same functional form has been successfully used to describe CdSe.^20^ In our case, we modified the original CdSe force-field (FF) to describe both CdSe and Cu_2_Se structures (see Methods for an accurate description of the model potential) using a single set of parameters. We generated CdSe matrices in both zb and wz phases by considering both bulk and nanowire structures depending on the specific calculation. In particular, the simulated NWs had a diameter of 4.5 nm and a length of 3 nm, while the bulk matrices were nonorthogonal supercells consisting of 2400 atoms. The long axis of the wz nanowires was aligned along the [001] crystallographic direction, while in the case of zb we consider the [111] direction. These orientations were specifically chosen to correspond to the growth directions of the Cdse nanowires experimentally investigated.

Since the specific NWs were carved out from bulk supercells, the resulting structures could be globally and locally charged. However, we observed that during the energy minimization procedure, the excess of positively charged Cd atoms was expelled from the nanowires, restoring the global neutrality.

#### Step (a): Formation of a Cu Thin Shell around a CdSe NW

**Figure 5:**
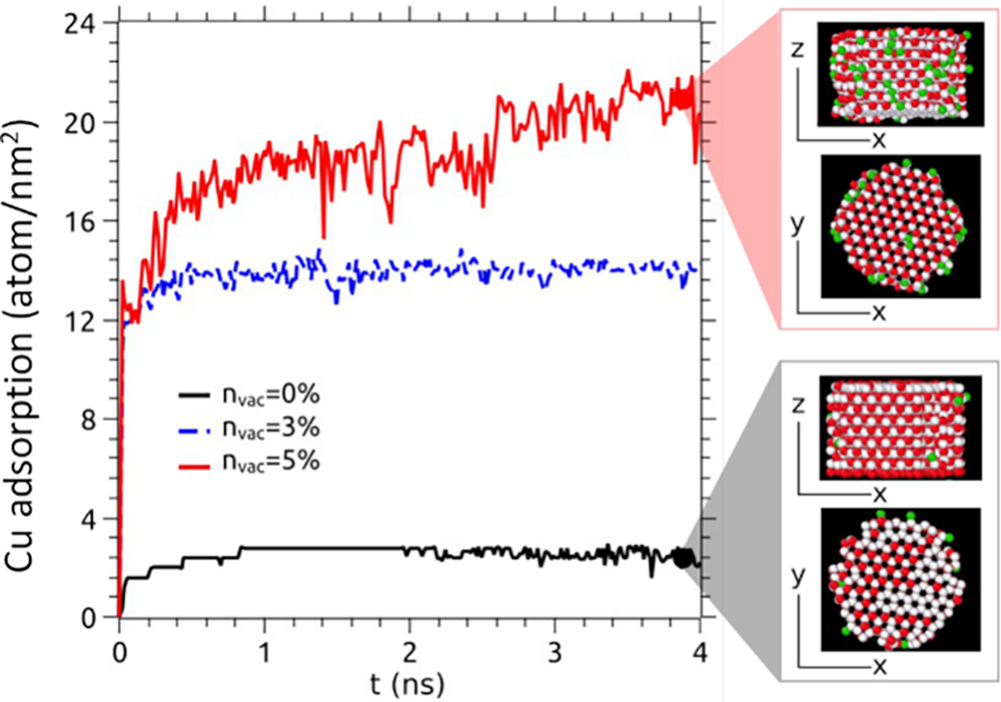
Amount of adsorbed Cu atoms per length unit on the CdSe nanowire with 5% (red), 3% (blue), and 0% (black) vacancy concentrations. Adsorbed Cu atoms tend to uniformly decorate the nanowire surface. The number of Cu atoms per unit length increases by increasing the CdSe NW vacancy concentration, which favors Coulombic attraction between free Cu atoms (green-colored) and the NW surface (red-colored atoms). The right panels show snapshots of the simulation at *t* = 1.4 ns showing the initial stages of creation of a Cu shell (green-colored atoms) on the NW viewed (red-colored atoms) in both top and frontal views.

In order to simulate the Cu adsorption on CdSe NWs with the creation of a Cu shell surrounding the nanowire, we aged the system composed of a CdSe nanowire embedded in a bath of 100 Cu atoms. We considered the case of a perfect wz CdSe nanowire, as well as the case of a nanowire containing small concentrations of Cd vacancies (below 5%). Based on the calculation of the formation energies for Cd vacancies (see Methods section), we expected a relatively low vacancy concentration (likely produced during the CdSe NWs synthesis). Such occurrence of a low Cd vacancy concentration was also experimentally observed in CdSe nanoparticles.^21^Figure 5 shows the concentration of Cu atoms adsorbed on the CdSe NW in the cases of 0% (black), 3% (blue), and 5% (red) vacancies.

The absorption of Cu atoms at the NW surface starts at the first steps of the MD simulations, reaching different saturation values after about 1 ns. We observe that a significant portion of the Cu atoms is adsorbed on the CdSe nanowires and that even a small concentration of Cd vacancies can significantly increase the fraction of adsorbed Cu atoms by a factor ≥ 5.

#### Step (b): Penetration of Cu Atoms into the CdSe Matrix and Subsequent Creation of a Cu Interstitial Defect in CdSe

After Cu adsorption on the NWs surface occurs, the spontaneous migration of Cu atoms into the NW is observed. More in detail, we defined a cylindrical region with the same axes of the nanowire and a radius of 1.7 nm, and we counted the number of Cu atoms entering it during the MD simulation, hereafter considered as the “core region” within the NW. After the adhesion of the first Cu atoms on the surface of the NW, a significant portion of them diffuses toward the core region, showing that Cu atoms spontaneously diffuse toward the nanowire center quite easily. The presence of Cu atoms in CdSe nanoparticles as substitutional defects was observed and analyzed using X-ray absorption spectroscopy.^22^

We quantified the energy needed for Cu diffusion from the surface to the core region by specifically considering the creation of Cu interstitial defects on CdSe upon Cu diffusion from the surface. More specifically, we calculated the corresponding energy landscape by taking into account as the initial and final structures a single Cu atom adsorbed at the CdSe surface and an interstitial Cu defect site, respectively, and assuming a straight diffusion path from the initial to the final Cu position, as depicted in panel b of Figure 4. In order to improve the statistics, we performed 30 different simulations where we changed the Cu initial positions along each Cartesian axis by a maximum value as large as 1 Å. The energy minimization was achieved by a combination of the Steepest-descend and Conjugate Gradients algorithms.

Figure SI_3 displays the estimated average energy landscape for wz (solid line) and zb (dashed line) CdSe matrices corresponding to the creation of an interstitial defect. A qualitative difference in the actual shape of the landscapes giving rise to corresponding different activation energies is apparent. We determined that, in the case of wz CdSe NWs, the actual Cu penetration from the surface into the bulk requires an energy barrier as large as *E*_step-b_ = 0.4 ± 0.1 eV, much higher with respect to the case of zb, which is *E*_step-b_ = 0.06 ± 0.01 eV. Therefore, we expect that, upon adhesion on the nanowire surface, a greater fraction of Cu atoms penetrates into the zb CdSe NWs than in the case of wz. This also gives a first indication about why copper is observed entering the zb CdSe NWs at *T* = 125 °C, while at the same temperature the phenomenon is not observed in wz CdSe. The difference in energy required for the Cu interstitial creation between wz and zb is attributed to their distinct surface reconstructions resulting from exposure to different surface orientations. Such surface reconstruction is largely extended in relatively small NWs. In particular, our observations revealed that the surface reconstruction in wz leads to a higher surface atom density compared to zb. This local increase in density next affects the migration of Cu atoms toward the inner region of the nanowire, thereby raising the energy barrier associated with the process.

#### Step (c): Diffusion of a Cu Interstitial Defect into the CdSe Matrix

We characterized the Cu diffusion process inside the CdSe matrix at different temperatures following the Cu penetration from the CdSe surface into the interstitial defect position. Aiming at disentangling any surface effects, we simulated Cu diffusion by considering bulk CdSe (in periodic boundary conditions). Finite temperature MD simulations were performed, where the trajectory of single Cu atoms for long time lapses (∼40 ns) was studied by estimating the mean square displacement (MSD). By linear fitting of the time evolution of the MSD, we extracted the corresponding Cu interstitial diffusion coefficient *D*(*T*) at different temperatures in the 600–2000 K temperature range and the corresponding migration energy *E*_step-c_ from the Arrhenius plots of the diffusion coefficient *D*(*T*) (see Figure SI_4). In the case of wz CdSe, a value of *E*_step-c_ = 0.29 ± 0.02 eV was obtained, while for zb CdSe a larger value of *E*_step-c_ = 0.51 ± 0.02 eV was determined, showing that, in the case of hexagonal CdSe, the Cu interstitial diffusion is more favorable than in the cubic phase.

Therefore, we expect that following the adhesion and the successive penetration of Cu atoms into the CdSe nanowire, the overall diffusion should be more favorable in the case of wz structures.

#### Step (d): Cu→ Cd Substitution via a Kick-Off Mechanism

We finally analyzed the last process described in our atomistic CE model, i.e., the Cd substitution by a Cu atom through a single kick-off mechanism. To this aim, we estimated the energy landscape using as initial configuration the CdSe NWs with one Cu interstitial defect, while the final configuration was obtained by swapping the interstitial Cu atom and the closest Cd atom, as shown in Figure SI_5. The corresponding energy landscape was then estimated by considering a straight path between the initial and final configurations. In order to improve the statistics, we performed 30 different simulations where we changed the Cu initial positions along each Cartesian axis by a maximum value as large as 1 Å.

Even in this case, we observed large differences between the energy barrier estimated for CdSe in the wz (*E*_step-d_ = 0.7 ± 0.3 eV) and zb (*E*_step-d_ = 0.3 ± 0.1 eV) phases, showing that in the latter case the Cu → Cd substitution via a kick-off mechanism is much more favorable than that in the former (Table 1).

**Table 1:** Energy Barriers and Migration Energy of the Three Different Processes Occurring in Both wz and zb CdSe and Corresponding to Copper Entering (Step b), Diffusing (Step b), and Replacing Cadmium (Step d)

	interstitial creation *E*_step-b_ (eV)	interstitial diffusion *E*_step-c_ (eV)	Cu → Cd kick-off *E*_step-d_ (eV)
wz CdSe	0.4 ± 0.1	0.29 ± 0.02	0.7 ± 0.3
zb CdSe	0.06 ± 0.01	0.51 ± 0.02	0.3 ± 0.1

The calculation of the corresponding activation energy was performed by assuming that the creation of a different stable configuration is taken as the step corresponding to a sudden energy decrease.

Based on the previous calculations of (i) the energy barrier *E*_step-b_ for the Cu penetration, (ii) the interstitial migration energy *E*_step-c_, and (iii) the energy barrier *E*_step-d_ for the kick-off mechanism, we eventually estimated the Boltzmann probability for each of the specific mechanisms previously investigated as exp(−*E*_step-b_/*k*_B_*T*), exp(−*E*_step-c_/*k*_B_*T*), and exp(−*E*_step-d_/*k*_B_*T*), respectively. By multiplying these three different probabilities the total CE probability (P_CE_) was estimated.

**Figure 6:**
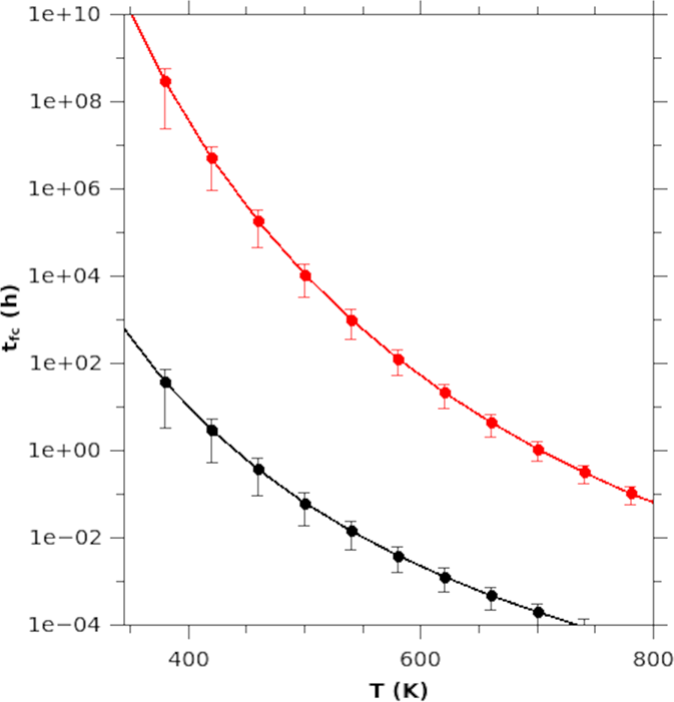
Estimated time required to observe the full CE conversion from a CdSe nanowire (diameter equal to *d* = 20 nm and length *l* = 150 nm) to a Cu_2_Se one, as a function of temperature for wz (red line) and zb (black line) starting CdSe crystal structure.

The time required to observe the full conversion of *N* = 76 × 10^4^ Cd sites (*t*_fc_) is shown in Figure 6 as a function of temperature, where *N* approximates the number of Cd atoms in both wz and zb single CdSe nanowires with a diameter *d* = 20 nm and length *l* = 150 nm, which are comparable to experimental data. We computed the expected full-conversion time as *t*_fc_=*N*/τ·*P*_CE_ where τ = 1/*f* and *f* is the attempted frequency for the CE thermally activated process. We guessed a value of *f* given by the Cu atom oscillation frequency around its potential energy minima, as shown in the energy landscape chart displayed in Figure SI_5. Thus, we evaluated *f* from a quadratic fit of the potential well, obtaining *f* = 1.9 THz, close to the frequency of an optical phonon in CdSe, around 3 THz. We considered CdSe with both zb and wz crystal structures, and with temperatures ranging in an interval comparable to the one experimentally observed, i.e., 300 K (27 °C) ≤ *T* ≤ 750 K (477 °C).

As expected, Figure 6 provides compelling evidence that *t*_fc_ depends on the system temperature for both crystals or, equivalently, the reaction is favored for both systems as the temperature is increased. It has also to be pointed out that our model predicts full-conversion times for the CE mechanism in rather good agreement with the experimental ones. In particular, the calculated CE rate of the investigated wz CdSe NW at 400 °C (673 K) is about 47 000 nm^3^/h. This is consistent with the CE rate of the *in situ* CE reaction reported in Figure 1A for a NW with diameter 18 nm at 400 °C, which is 49 000 nm^3^/h. Moreover, this result can also be put in relation with our reported experiments,^18^ where thinner wz CdSe NWs were used. In fact, a full CE was obtained in 30 min over the whole volume of length of 350 nm for a WZ CdSe NWs, which corresponds to a CE rate of 55 000 nm^3^/h for the *in situ* CE reaction performed at 400 °C. Even though in this case the real exchanged wz CdSe NW was thinner and longer than the calculated one (10 nm in diameter and 350 nm in length versus 20 nm in diameter and 150 nm in length, respectively), the two volumes are comparable, with the calculated NW being slightly bigger than its experimental counterpart. Then, the slight difference in time required for a full conversion in the two cases sounds reasonable and there is a good agreement with the experimental CE rates, with the slightly faster rate of the thin NW likely caused by its higher surface-to-volume ratio.

Moreover, using our atomistic model, we can predict the occurrence of a complete CE mechanism at significantly lower temperatures in the case of zb CdSe with respect to wz, once again in full agreement with the experimental results. We attribute such a difference to the activation energy for the Cu → Cd kick-off mechanism, as it is significantly lower in the case of zb CdSe than in that of wz. We remark that, among the three different identified mechanisms, in the case of wz CdSe, the Cu → Cd kick-off is by far the bottleneck for the whole process having a probability significantly lower with respect to both Cu diffusion and Cu penetration. Conversely, in the case of zb CdSe, the bottleneck step is represented by Cu diffusion into the CdSe matrix. This result also agrees with the experimental evidence. Indeed, if we consider that the typical duration of an *in situ* (S)TEM CE experiment is below 10 h, the temperature required for the full exchange of the NW volume for a CdSe NW of fixed dimensions in the zb phase is much smaller (i.e., the calculated CE rates are higher) than that for the wz one.

One possible alternative path for CE involves the generation and, next, the migration of Cu interstitials within the Cu_2_Se domain (rather than within the CdSe domain), assisted by a kick-off mechanism occurring at the interface between CdSe and Cu_2_Se.

In order to investigate the validity of such a possible mechanism, we have undertaken a comprehensive analysis focusing on the creation and diffusion of interstitial particles within the Cu_2_Se domain. Initially, we examined the energy cost associated with the Cu interstitial creation in a Cu_2_Se nanowire with a diameter of 4.5 nm, a length of 3 nm, and the [001] crystallographic orientation corresponding to the long axis of the NW. By considering a single Cu atom adsorbed on the Cu_2_Se surface as the initial structure and an interstitial Cu defect site as the final one, we calculated the energy landscape for this transition. We assumed a direct diffusion path from the initial position of Cu to the final one. The estimated average (averaged over as many as 30 different reaction paths having the same two sites) energy landscape for this interstitial creation process is depicted in Figure SI_6. Notably, our findings demonstrate a considerably higher energy barrier (0.6 ± 0.2 eV) as compared to CdSe (both wz and zb) case. This observation suggests that step b (Cu interstitial creation) acts as a bottleneck for CE. Consequently, our analysis leads us to the conclusion that the comparatively high energy required for the creation of Cu interstitials in a Cu_2_Se nanowire, in comparison to that in CdSe, makes the mechanism of interstitial creation and diffusion within the Cu_2_Se domain less likely. Instead, the direct cation exchange mechanism occurring within the CdSe nanowires is favored over the formation of interstitial defects in Cu_2_Se and the subsequent migration into the CdSe domain.

To achieve a more accurate description of the CE mechanism, it would be necessary to explicitly represent it as a diffusion process, employing Fick’s law in the modeling, which would allow us to incorporate the effects of varying geometries, such as rods versus spheres. However, implementing Fick’s law in an MD simulation would require explicit observation of the CE mechanism, leading to simulation times that are currently beyond the computational capabilities. Accordingly, the analysis provided in our paper must be intended as an order-of-magnitude estimation.

Moreover, the easier kick-off observed in calculated CE reactions for zb CdSe NWs can be exploited to understand the transient reverse-CE experimentally observed solely in smaller exchanged domains, as shown in Figure 2. Here the Cu_2_Se exchanged portions of NW can be divided between smaller domains, roughly 5 nm long (left and right sides of the NW), and bigger domains, approximately 20 and 30 nm long (central portion of the NW). Increasing the temperature, and consequently the kinetics of CE, three paths are observed based on the actual size: the smaller domains become shorter (around 3 nm) until they disappear, while the 20 nm long domain moves slightly along the NW with limited growth and the 30 nm long domain grows until merging with the 20 nm domain. Thus, aiming at investigating the microscopic origin of such selective behavior, we performed additional MD simulations on three different model systems, whose sizes were made as large as possible to be comparable to the experimentally observed phenomena, still requiring an affordable computational workload. The results of these simulations are reported in the Supporting Information.

## Conclusions

In this work, we expanded our previous *in situ* TEM studies on thermally activated CE reactions in the solid state between populations of cubic Cu_2_Se NCs and hexagonal CdSe NWs. Here, we used a MEMS-based *in situ* heating holder, a more resolved STEM-based imaging strategy. Additionally, the Cu_2_Se NCs were also paired with cubic CdSe NWs to verify how the crystal structure of the acceptors influences the CE reaction. Through these experiments, we observed a common, general trend in CE reactions whose threshold temperatures and evolution change with the crystalline phase of the host matrix. In particular, we observed that copper expulsion starts below 125 °C and that expelled copper forms an external shell around the CdSe NWs before giving rise to CE. Its diffusion in the CdSe matrix and the Cd replacement occur at 400 °C for hexagonal CdSe NWs and at 125 °C for cubic CdSe NWs (Video_S1). We also found the existence of an unstable regime up to 250 °C, where the smaller domains of Cu_2_Se in the NWs can be transformed back into CdSe domains while the bigger ones keep growing in length. Eventually, the CE reaction was simulated using a classical MD approach, providing numerical estimations of the activation energies for each subsequent step, and determining the probabilities of the whole CE reaction for the different CdSe crystal structures. In addition, the MD simulations contributed to explaining the partial reversibility of Cu_2_Se smaller domains into CdSe observed in the cubic NWs. The combination of experimental and theoretical approaches provides a solid framework for imaging and interpreting thermally activated CE at the solid state, but also offers a set of results of general interest by showing the mechanisms that command the onset of CE reactions with a much higher level of detail and demonstrating how varying the crystal structure of the nanoparticles involved determines a drastic variation in the environmental condition and unfolding of the whole CE reaction.

## Methods

### Experimental Section

#### Chemicals

Oleylamine (OLAM, 70%), oleic acid (OLAC, 90%), 1-octadecene (ODE, 90%), toluene (>99%), ethanol (99.8%), and acetone (99.5%) were all purchased from Sigma-Aldrich and used as received. Copper chloride (CuCl, 99.999%), bismuth chloride (BiCl_3_, >98%), cadmium oxide (CdO, 99.99%), selenium (Se 99.99%), trioctylphosphine oxide (TOPO, 99%), and trioctylphosphine (TOP, 97%) were purchased from Strem Chemicals.

### Materials Synthesis and Characterization

#### Synthesis of zb CdSe Nanowires

The CdSe nanowires were prepared by following a previously reported method called as solution–liquid–solid (SLS) growth.^23^ In this method, *in situ* formed metal Bi nanoparticles in solution were used as the catalyst to initiate the growth of solid CdSe nanowires. In a typical synthesis, a mixture of CdO (50 mg, 0.4 mmol), OA (0.5 mL, 1.6 mmol), and TOPO 99% (5.0 g, 12.9 mmol) was first loaded in a 3-neck flask and heated to 120 °C to liquefy the TOPO power. The above mixture was degassed at 120 °C for 1 h and then was heated to 320 °C until the CdO was dissolved. The obtained solution was decreased and maintained at 250 °C for the growth. Meanwhile, an injection solution containing the Se source (0.1 mL, 1 M TOPSe, Cd/Se ratio = 4:1) and Bi precursor (0.03 mL, 2 mM BiCl_3_ in acetone) was prepared in the glovebox. After the injection, the reaction was allowed to proceed for 5 min and then stopped by removing the heating mantle; 10 mL of toluene was added when the temperature was lowered to 120 °C. The obtained nanowires were precipitated from solution by centrifuging directly and then dispersed in toluene. We found that the obtained nanowires tend to aggregate upon additional washing, which is different from that of the colloidal quantum dots.

#### Synthesis of wz CdSe Nanowires

The wz nanowires were prepared following the method already reported in ref (18). In particular, the samples were obtained by slightly modifying a published procedure.^23^ A mixture of CdO (75 mg), oleic acid (750 μL), and TOPO (7.5 g) was placed in a 3-neck 50 mL round-bottom flask and degassed under vacuum at a temperature of 120 °C for 1 h. The flask was then backfilled with nitrogen, and the temperature was raised to 320 °C to facilitate the formation of the Cd-oleate complex, characterized by the transformation of the red suspension into a transparent solution. The temperature was lowered to 250 °C, and a mixture of Se:TOP (75 μL; 1 M of Se in TOP) and BiCl_3_ in acetone (37.5 μL; 2 mM of BiCl_3_ in acetone) was swiftly injected. The reaction mixture was allowed to stir for 2 min at this temperature, and the heating mantle was removed thereafter to allow the solution to cool down. A volume of 5 mL of toluene was added, and the nanowires were washed with methanol. Subsequent washing was performed with toluene alone to remove the undesired spherical CdSe NCs formed during the course of the reaction, aided by the fact that the heavier nanowires settle at lower centrifugation speeds. The nanowires were redispersed in toluene.

#### Synthesis of Cu_2_Se Nanocrystals

The Cu_2_Se nanocrystals were prepared according to a method reported by us.^24^ In a typical synthesis, a mixture of anhydrous CuCl (0.099 g, 1 mmol), 5 mL of OLAM, and 5 mL of ODE was loaded in a reaction flask and degassed at 100 °C for 1 h. The above solution was then heated to 300 °C. Meanwhile, an injection solution containing selenium powder (39 mg, 0.5 mmol) and 3 mL of oleylamine was prepared in the glovebox. After injection, the reaction was allowed to proceed for 15 min and stopped by removing the heating mantle. The obtained nanocrystals were precipitated from the solution by adding ethanol and centrifuging.

#### TEM/STEM Characterization

Spherical aberration (Cs) corrected high resolution transmission electron microscopy (HRTEM), in scanning mode (STEM/HRSTEM), along with energy-dispersive X-ray spectroscopy (EDS) and electron energy loss spectroscopy (EELS) elemental mapping, was carried out on a double spherical corrected FEI Titan Themis microscope, equipped with an ultrabright Schottky (XFEG) electron source, a S-Twin objective lens, a SuperX EDS spectrometer with a 0.7 srad collection angle, a Gatan Quantum EELS image filter, and a FEI Ceta complementary metal oxide semiconductor (CMOS) camera. The microscope operated at an acceleration voltage of 300 kV, with an ultimate resolution of 0.6 Å in STEM mode. Structural characterization was performed by analyzing the two-dimensional fast Fourier transform (2D-FFT) numerical diffractograms and measuring the planar and angular relationships occurring between diffraction spots. The *in situ* heating was performed by a MEMS-based Dens Solution Wildfire-series TEM/STEM specimen holder, using chips with carbon film support for heating the mixture of Cu_2_Se and wz CdSe NWs, and with silicon nitride support for heating the mixture of Cu_2_Se with zb CdSe NWs. Due to the expected superposition of the nitrogen EELS K edge (401 eV) with the Cd M_4,5_ one (404 eV), this further determined the choice to exploit STEM-EDS instead of STEM-EELS for chemically mapping the mixture of Cu_2_Se NCs with zb CdSe NWs. The MEMS where the mixture of Cu_2_Se NPs and wz CdSe NWs was deposited were shortly cleaned with ethanol and subsequently heated at 130 °C for 10 min upon vacuum conditions in a Gatan 655 turbo pumping station before inserting them in the TEM column. The MEMS where the mixture of Cu_2_Se and zb CdSe NWs was deposited were shortly cleaned with ethanol without additional heating to avoid the CE reaction from starting before the insertion in the microscope.

### Theoretical Section: Molecular Dynamics Simulations

#### Force-Field Development for CdSe and Cu_2_Se

All the calculations presented in the atomistic CE model have been performed by means of classical molecular dynamics using a suitable force-field (FF) able to reproduce the mutual interaction between Cu atoms and CdSe both in the wurtzite as well as zincblende phases. Based on the FF already developed for CdSe, we developed our specific FF by defining the mutual Cd–Cu interactions in order to properly describe the lattice constants of Cu_2_Se by keeping fixed the original set for CdSe.

In particular, our FF consists of a combination of a Lennard-Jones plus an electrostatic term as
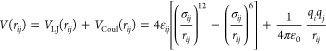
2where *r*_ij_ is the interatomic distance between atom *i* and atom *j*, *ϵ*_*ij*_ is the depth of the potential well, σ_*ij*_ is the distance at which the potential energy is zero, and *q*_*i*_ and *q*_*j*_ are the atomic partial charges.

The parameters taken from^20^ (shown in Table 2) well describe the lattice constants, angles and other structural parameters as the elastic constants of CdSe.

**Table 2:** Parametrization for CdSe^a^

LJ+Coul parameters	value
ε_CdCd_	1.68 meV
ε_SeCd_	1.58 meV*
ε_SeSe_	1.49 meV
σ_CdCd_	1.98 Å
σ_SeCd_	3.61 Å*
σ_SeSe_	5.24 Å
*q*_Cd_	+1.18|e|
*q*_Se_	–1.18|e|

a Values with an asterisk (*) are obtained by following the Lorentz–Berthelot mixing rules.

It is important to notice that the cross terms involving two different atomic species are calculated following the usual Lorentz–Berthelot mixing rules.

Using the same functional form, we extended the parameter set in order to describe Cu_2_Se by first setting the specific atomic Cu partial charge coherently with that of Se: *q*_Cu_ = 0.59|e| (we account for one atom of Se by two of Cu) by maintaining the system neutrality.

The remaining 4 + 2 parameters (including the Cd–Cu interaction) have been selected by applying the combination rules to the cross terms in Table 2. Finally, σ_CuCu_ and ε_*CuCu*_ have been obtained by fitting the lattice parameters of the material, obtaining the final set in Table 3.

**Table 3:** Fitted Parameter Set Including CuCd Interactions^a^

LJ+Coul parameters	value
ε_CuCu_	1.20 meV
ε_SeCu_	1.33 meV*
ε_CdCu_	1.42 meV*
σ_CuCu_	1.00 Å
σ_SeCu_	3.12 Å*
σ_Cd_	3.61 Å*
q_Cu_	+0.59 |e|

a Values with an asterisk (*) are obtained following the Lorentz–Berthelot mixing rules.

#### Validation of the Force Field

The structural parameters of wurtzite CdSe and antifluorite Cu_2_Se obtained from our FF are listed in Table 4 together with the corresponding values calculated using DFT showing generally good agreement.

**Table 4:** Structural Parameters Used to Check the Goodness of Our Parameter Set

		published experimental value	published DFT value	reported DFT value	reported FF value
wurtzite-CdSe	latt. param.	*a* = 4.30 Å^25^	*a* = 4.29 Å^26^	*a* = 4.33 Å	*a* = 4.39 Å
		*c* = 7.01 Å^25^	*c* = 7.01 Å^26^	*c* = 6.87 Å	*c* = 7.02 Å
			*u* = 0.376^26^	u = 0.385	*u* = 0.40
wurtzite-CdSe	cell angles		α = 90°^26^		α = 90°
			γ = 120°^26^		γ = 120°
antifluorite-Cu_2_Se	latt. param.	*a* = 5.859 Å^27^	*a* = 5.80 Å^28^	*a* = 5.603 Å	*a* = 5.666 Å
antifluorite-Cu_2_Se	cell angles		α = 90°^28^		α = 90.07°

Further validation of our FF is obtained by specifically analyzing not only the specific Cd–Se and Cu–Se interactions but also the Cd–Cu interaction. To this aim, we performed a campaign of DFT and MD simulations to describe the mutual interactions of all three species. In particular, we focus on the description of Cu adsorption on CdSe NWs by comparing the Cu/CdSe attraction basin estimated with our FF with the one estimated using DFT, as shown in Figure SI_12.

The DFT calculations were performed by considering two different NW radii by obtaining similar results. We observe an overall qualitative agreement between FF and DFT. In both cases, we observe an energy-deep basin to trap a Cu atom close to the nanowire surface at room temperature (at a distance *d* = 1.8 Å). This result agrees with the experimental findings of the formation of a Cu shell around the CdSe structures. However, a more quantitative comparison between FF and DFT shows that DFT provides an attraction basin deeper by a factor of 3 than the one obtained with the FF.

Further validation of the FF was performed by estimating the formation energies for specific defects. The formation energy for a specific kind of defect is computed for different cell sizes. In Figure SI_13, the formation energies of a Cd vacancy in CdSe are shown as a function of the number of atoms in the computational cell.

The formation energies for both the Cd vacancy and Cu interstitial defect in the CdSe crystal are listed in Table 5.

**Table 5:** Formation Energies for the Cd Vacancy and Cu Interstitial Defect in CdSe

	DFT formation energy (eV)	FF formation energy (eV)
Cd vacancy in CdSe	4.9	5.3
Cu interstitial in CdSe	0.4	1.0

## Supporting Information Available

Intensity profiles recorded perpendicularly to the length of the NW on the Cu EELS and EDS elemental maps of Figure 1B and 2; representative HRTEM images of a zb CdSe NW recorded along its length; results of theoretical simulations ancillary to those reported in the main text, such as average energy landscape for a straight diffusion path from the initial surface position to the final interstitial position of a Cu atom for wz and zb CdSe matrices; estimation of diffusion coefficient for interstitial defect in a wz and zb CdSe matrix at diverse temperatures; average energy landscape corresponding to a straight path of a single Cu atom from a CdSe interstitial position to a Cd substitutional position, obtained via a kick-off mechanism; average energy landscape corresponding to a straight diffusion path from the initial surface position to the final interstitial position of a Cu atom for Cu_2_Se; MD simulations results (text and figures) on three different model systems (MS1, MS2 and MS3) aiming at explaining appearance and disappearance of the lower-sized Cu_2_Se domains in zb CdSe NWs with the thermal increase; attraction basin for a Cu atom close to a CdSe NW, determined by both FF and DFT; Cd vacancy formation energy estimated using DFT and FF (PDF)
Video animation summarizing what happens when a zb CdSe NW encounters copper atoms expelled by Cu_2_Se NCs as a consequence of the thermal stimulus (MOV)

## Supplementary Material

nn3c04516_si_001.pdf

nn3c04516_si_002.mov

## Abbreviations

CE: cation exchange
DFT: density functional theory
EDS: energy dispersive X-ray spectroscopy
EELS: electron energy loss spectroscopy
FF: force field
HRSTEM: high resolution scanning transmission electron microscopy
HRTEM: high resolution transmission electron microscopy
MD: molecular dynamics
MEMS: microelectronic mechanical systems
MSD: mean square displacement
NC(s): nanocrystal(s)
NR(s): nanorod(s)
NW(s): nanowire(s)
STEM: scanning transmission electron microscopy
TEM: transmission electron microscopy
2D-FFT: 2-dimensional fast Fourier transform
wz: wurtzite
zb: zincblende.
