# Supplementary material for: Thermally Promoted Cation Exchange at the Solid State in the Transmission Electron Microscope: How It Actually Works
Source: ACS Nano. 2023 Aug 28;17(17):17058–69. doi: 10.1021/acsnano.3c04516 (PMC10510578; doi:10.1021/acsnano.3c04516)
Supplement: Supplementary file 1 — nn3c04516_si_001.pdf [file nn3c04516_si_001.pdf]

Supporting Information

Thermally Promoted Cation Exchange at the Solid State in the Transmission Electron Microscope: How It Actually Works

Alberto Casu[‡,†], Miquel Lopez[§], Claudio Melis[§], Davide Deiana[#], Hongbo Li[$], Luciano Colombo[§], and Andrea Falqui[‡,†,@,]*

[‡] University of Milan, Department of Physics "Aldo Pontremoli", Via Celoria 16, 20133 Milan, Italy

[†] King Abdullah University of Science and Technology (KAUST), Biological and Environmental Sciences and Engineering (BESE) Division, Nabla Lab, Thuwal 23955-6900, Saudi Arabia

[§] Department of Physics, University of Cagliari, Cittadella Universitaria di Monserrato, 09042 Monserrato (CA), Italy

[#] Centre Interdisciplinaire de Microscopie Électronique (CIME), Ecole Polytechnique Fédérale de Lausanne (EPFL), 1015 Lausanne, Switzerland

[$] Experimental Center of Advanced Materials, School of Materials Science and Engineering, Beijing Institute of Technology, Beijing 100081, China

[@] Interdisciplinary Centre for Nanostructured Materials and Interfaces (CIMaINa), Department of Physics "Aldo Pontremoli", University of Milan, Via Celoria 16, 20133 Milan, Italy

[*] *Corresponding author: Andrea Falqui, e-mail: andrea.falqui@unimi.it*

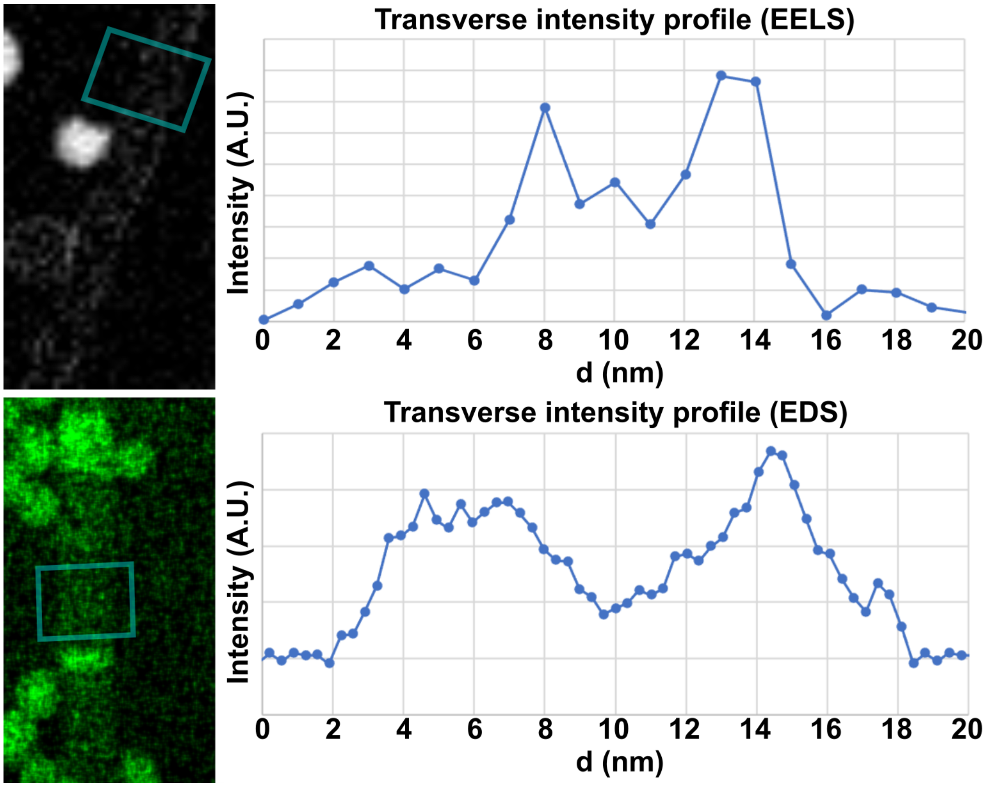

Figure SI_1. Averaged intensity profiles recorded along the width (*i.e.*, the direction perpendicular to the length of each NW) of the non-exchanged zones of CdSe NWs. The panel at the top refers to an EELS map also presented in Figure 1B for a wz CdSe NW, the panel at the bottom refers to a closeup of an EDS map also presented in Figure 2 for a zb NW. The pale blue rectangles indicated the portions of NWs used to collect each profile.

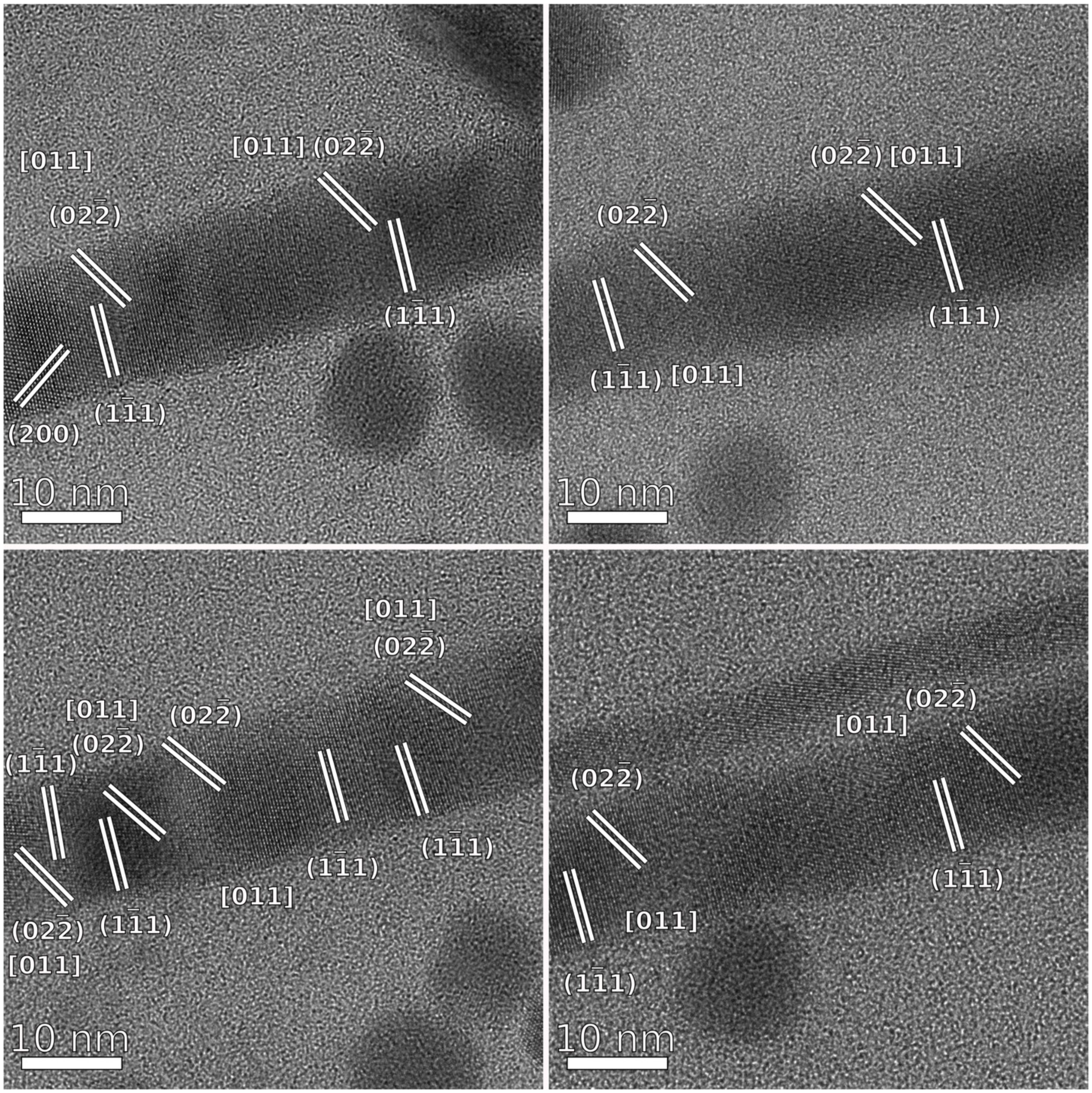

Figure SI_2. Representative HRTEM images collected along the length of a zb CdSe NW. Interplanar distances and angular relationships are consistent with zb CdSe (JCPDS card no. 190191). Repeated sets of planes in each panel indicate the presence of distinct crystalline domains.

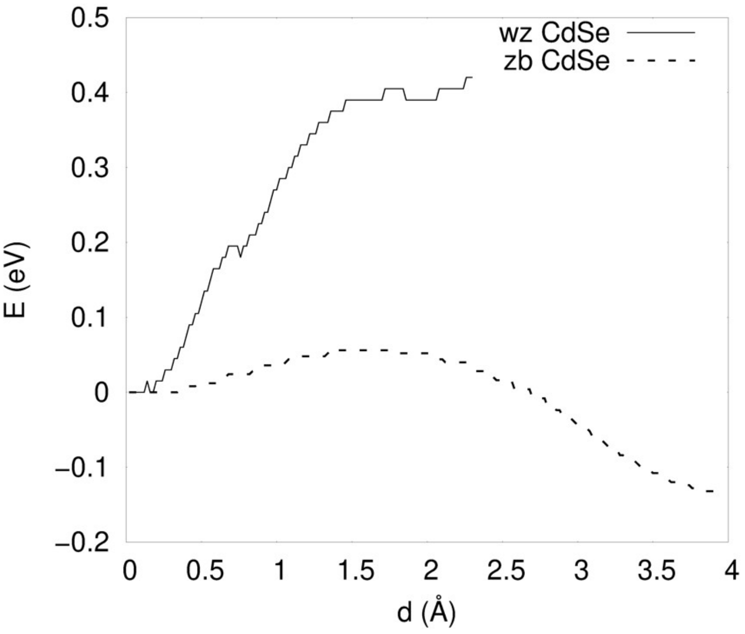

Figure SI_3. Average energy landscape corresponding to a straight diffusion path from the initial surface position to the final interstitial position of a Cu atom for wz (solid line) and zb (dashed line) CdSe matrices.

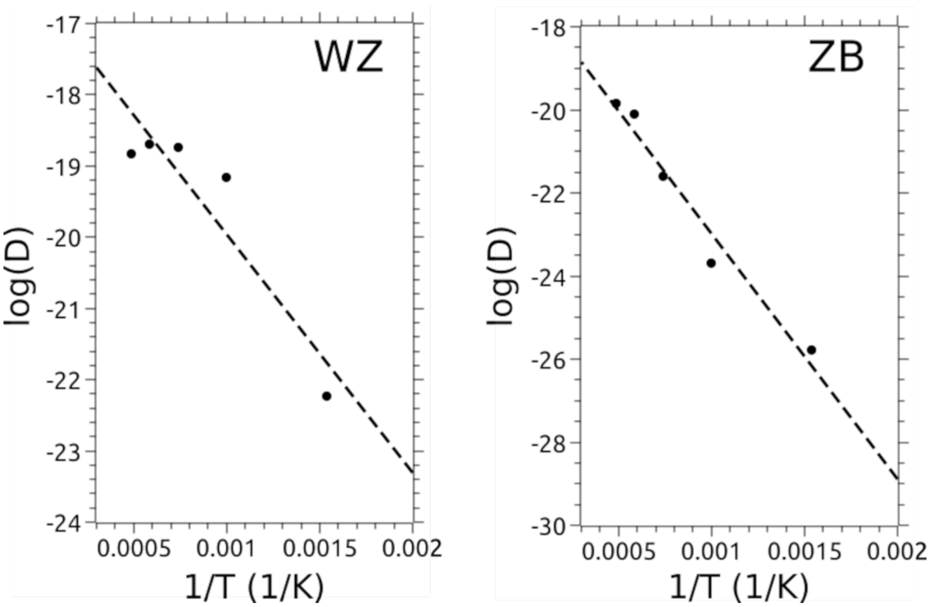

Figure SI_4. Diffusion coefficient D(T) estimated for the Cu interstitial defect in a wz (left) and zb (right) CdSe matrix at different temperatures. The time-lapse used for each time series is $40 \cdot 10^6$ simulation steps.

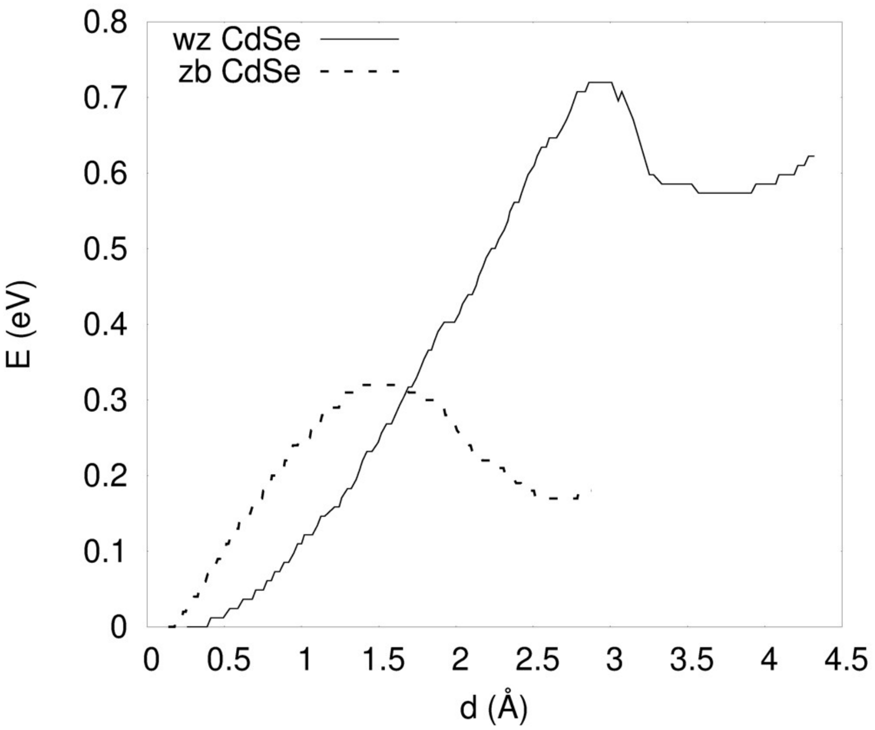

Figure SI_5. Average energy landscape corresponding to a straight path of a single Cu atom from a CdSe interstitial position to a Cd substitutional position obtained via a kick-off mechanism.

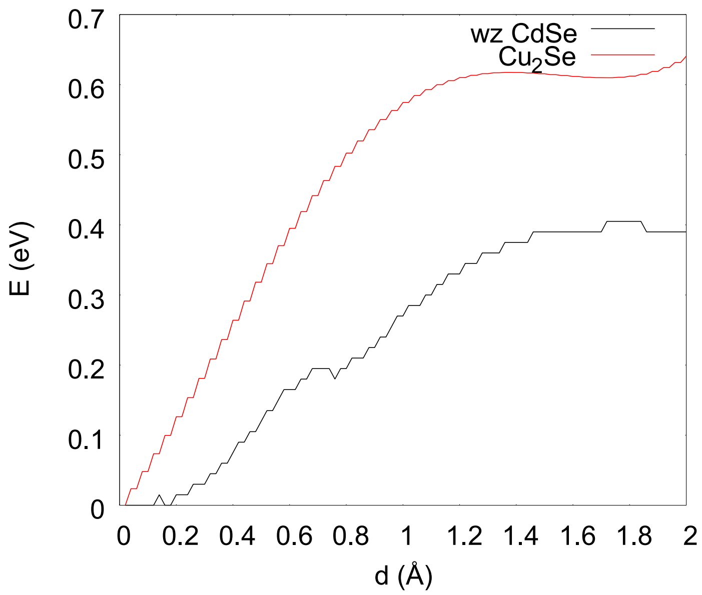

Figure SI_6. Average energy landscape corresponding to a straight diffusion path from the initial surface position to the final interstitial position of a Cu atom for Cu_2Se.

MD Simulations performed to investigate the evolution (formation and possible disappearance) of the Cu_2Se domains during the thermal increase in the zb CdSe nanowires

We investigated the following systems:

Model system 1 (MS1): zb CdSe NW (with total length L_{TOT}=5 nm and radius r=5 nm where a small (L_R=1 nm) Cu_2Se region (see Fig. SI_7, left) was centrally inserted.

Model system 2 (MS2): zb CdSe NW with total length L_{TOT}=10 nm and radius r=5 nm where a large (L_R=6 nm) Cu_2Se region (see Fig. SI_7, middle) was inserted.

Model system 3 (MS3): zb CdSe NW with total length L_{TOT}=15 nm and radius r=5 nm where both small and a large Cu_2Se (with size L_{R1}=1 and l_{R2}=4 nm, respectively) were inserted (see Fig. SI_7, right).

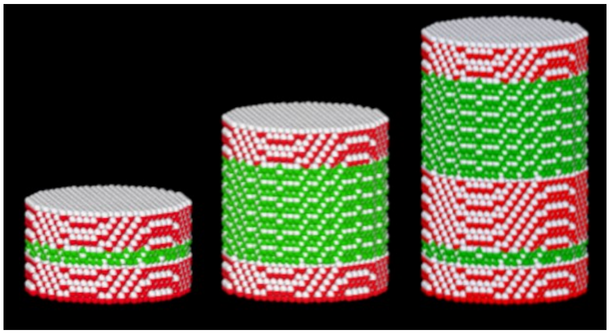

Figure SI_7. Representation of the three CdSe/Cu_2Se models systems investigated by classical MD. Left: MS1, middle: MS2 and right: MS3. Cadmium, selenium, and copper atoms are represented in red, white, and green color, respectively.

After optimizing the geometry by a combination of steepest-descent and conjugate-gradients algorithms, we performed 25 ns-long MD simulations at T=750 K. The choice of such a temperature, larger than the experimental one, was made for accelerating the actual CE kinetics, without largely affecting the system stability (we stress that in this case we did not aim at calculating rates; rather, we simply focused on atomic-scale structural evolution). As well, to reduce the complexity of the simulated systems, we did not consider any external Cu atom positioned around the nanowire as well as the presence of Cd vacancies in the nanowire. The outcome of the MD simulations of the three systems can be rationalized as follows.

MS1. To investigate the diffusion of Cu and Cd atoms during the whole 25 ns dynamics of the MS1, we estimated the atomic radial distribution (*i.e.*, the number of atoms divided by corresponding ring surface area with radius R) in the NW (see Fig. SI_8, left panel) at time t=0, 12.5 and 25 ns. While the Cd radial distribution appeared almost unaffected during the dynamics, we observed a significant variation in the case of Cu: we observed a clear evolution from an initially almost flat distribution towards a final distribution characterized by two peaks at Rcoordinate ~0 and 4.5 nm.

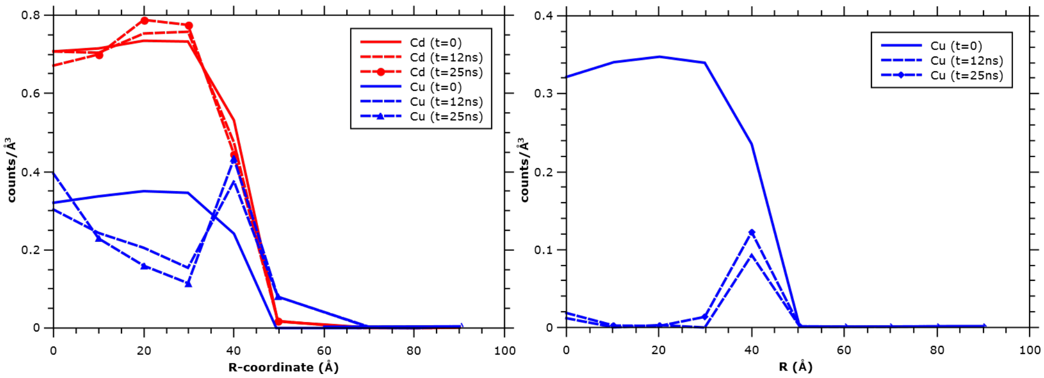

Figure SI_8. MS1(left panel): starting and following evolution of Cu (blue atoms) and Cd (red atoms) radial distribution at increasing times (0, 12 and 25 ns). Right panel: Cu radial evolution in a thin portion of MS1 containing only the Cu_2Se domain with a thin layer of zb CdSe on its top.

The first spurious peak is amplified by the small value of the ring surface area, while the second peak at R ~4.5 nm with a value of 0.4 atoms/Å^3 is associated with the Cu diffusion towards the NW surface. In that case, we were further and strongly interested in discerning whether Cu atoms

diffuse into the CdSe part and/or if they are expelled out of the NW directly from the Cu_2Se region. With this aim, we limited the calculation of the Cu radial distribution only to the Cu_2Se region (see Fig SI_8, right panel) and observed the presence of the second peak at R ~4.5 nm with a smaller value of 0.15 atoms/Å^3, concluding that the Cu_2Se domain inserted into a larger CdSe region tends to disappear due to the diffusion of the Cu atoms towards the NW surface both in the Cu_2Se and CdSe regions. The top row of Fig. SI_9, (a top snapshot of the NW at the same time intervals) makes apparent how Cu (green) atoms tend to migrate towards the NW surface during the CE.

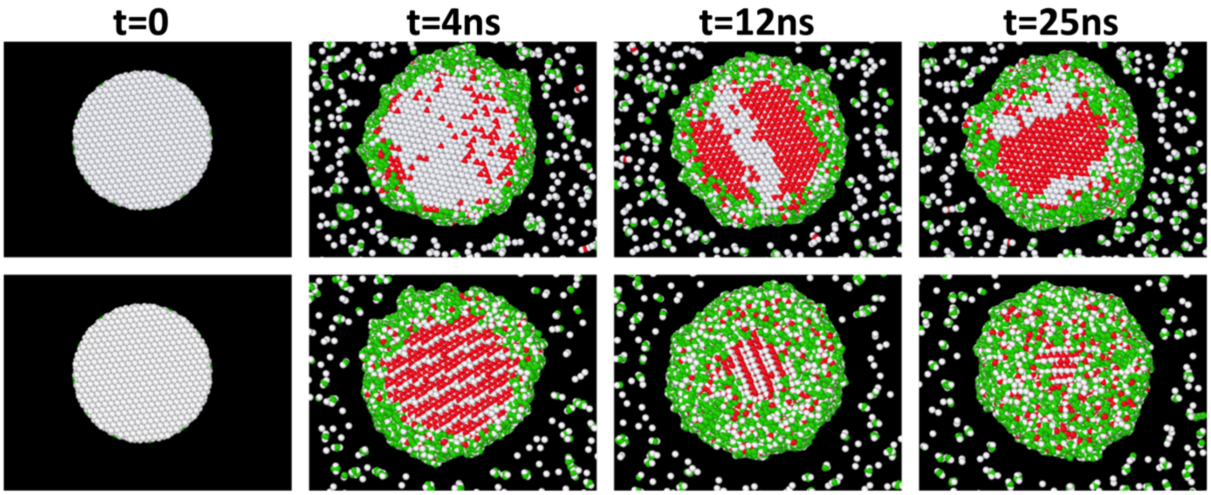

Figure SI_9. Top row: top view of the NW evolution in MS1 during the 25 ns dynamics. Bottom row: top view of the NW evolution in MS2 during the same dynamics.

MS2. When the Cu_2Se domain was made larger, we observed large differences in the Cu radial distribution with respect to MS1. Indeed, the occurrence of the peak at R-coordinate ~4.5 nm in Cu radial distribution (see Fig. SI_10, left panel) is no longer observed.

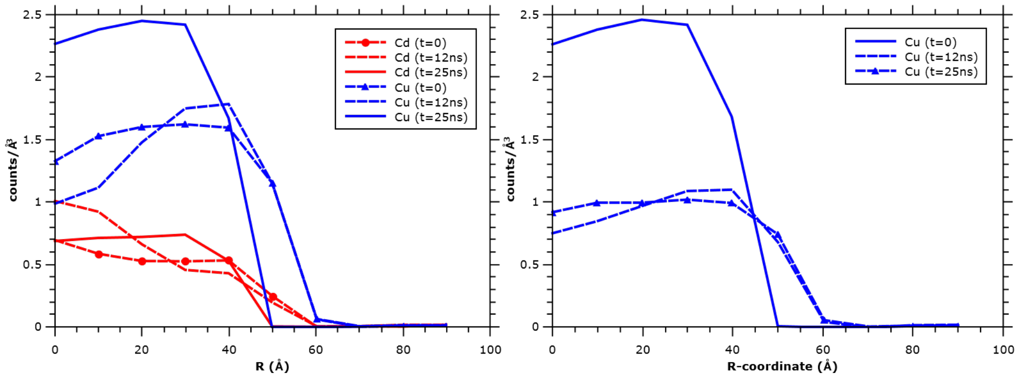

Figure SI_10. MS2 (left panel): starting and following evolution of Cu (blue atoms) and Cd (red atoms) radial distribution at diverse times (0, 12 and 25 ns) of the accelerated dynamics. Right panel: sole Cu radial evolution in a portion of MS2 containing only a Cu_2Se domain with a thin layer of zb CdSe on its top.

The analysis of the radial distribution focused only on the Cu_2Se region confirms that Cu atoms do not massively move towards the NW surface, but instead tend to uniformly distribute on the whole NW including the CdSe domain. A more visual idea of what happens in that case can be obtained by the bottom row of Fig. SI_9, where the Cu diffusion towards the whole NW during the CE dynamics is clearly shown. These results suggest that relatively large Cu_2Se sections (*i.e.*, with size larger than ~5nm) are not reabsorbed by the CdSe regions during the dynamics, but instead tend to endure and then expand on the whole NW.

MS3. Finally, we performed comparable MD simulations for MS3, where a small and large Cu_2Se domain initially coexist. The analysis of the Cu radial distribution (see Fig SI_11) confirms that in this case the Cu atoms belonging to the small Cu_2Se domain diffuse towards the NW surface leading to the domain disappearance. Conversely, the larger domain displays a radial distribution

peak corresponding to the NW surface, thus confirming that in this case Cu atoms diffuse towards the CdSe domain leading to an expansion of Cu_2Se on the whole NW.

The stability or the disappearance of Cu_2Se domains within the CdSe phase can be explained in terms of capillarity effects. The mechanism driving the formation and growth of an *a*-phase crystal within a *b*-phase matrix can be described through the following thermodynamic equation:

$$PdV \quad = g_{\mathrm{ab}}dV - \gamma_{\mathrm{ab}}dS \quad (1)$$

In this equation, P is the thermodynamics pressure acting at the a-c boundary, dV is the transformed volume, g_{ab} represents the free energy difference between the *a* and *b* phases, while γ_{ab} describes the free energy associated with the surface reconstruction of the *b*-phase crystal within the *a*-phase matrix. Significantly, a specific *b*-seed can be considered stable when the value of P is greater than zero ($P > 0$). Thus, the threshold conditions for the stability vs. disappearance of such a seed can be defined by determining the specific values of dS and dV that result in $P = 0$.

When a *b*-seed is stable, its size undergoes growth at a rate that eventually reaches a constant value determined by g_{ab}, namely the free energy difference between the *a*- and *b*-phases, along with a mobility factor dictated by various material parameters. However, if local thermodynamics is such that the *b*-seed is not stable, it will tend to diminish and convert back into an *a*-phase.

Based on the preset model, it is possible to estimate the the minimum radius R* of a stable *b*seed inside the *a*-phase matrix: $R^*=R|_{P=0}=2\gamma_{ab}/g_{ab}$.

The values for g_{ab} and γab are not easy to calculate or measure experimentally, and unfortunately, there is no literature available on these values for a Cu_2Se seed growing in a CdSe matrix. However, we can make an initial assumption that the energy required for the surface reconstruction of a Cu_2Se surface in CdSe is the same as the energy required for a Cu_2Se surface in a vacuum.

Using this assumption, we can estimate the free energy associated with surface reconstruction at

T=673 K to be $\gamma_{ab,673\ K}$= 0.320 eV/Å^2.

Additionally, based on Ref. [18], we can estimate the free energy difference g_{ab} between wz CdSe and a Cu_2Se crystal at T=673K to be $\Delta G_{ab,673\ K}$= 0.0219 eV/Å^3.

Using these values, we can estimate the minimum radius a Cu_2Se-seed needs to be energetically stable at T=673 K to be $R^*=R|P=0=2\gamma_{ab}/g_{ab}$=2.9 nm. This estimate is consistent with both the experimental results and the MD simulations.

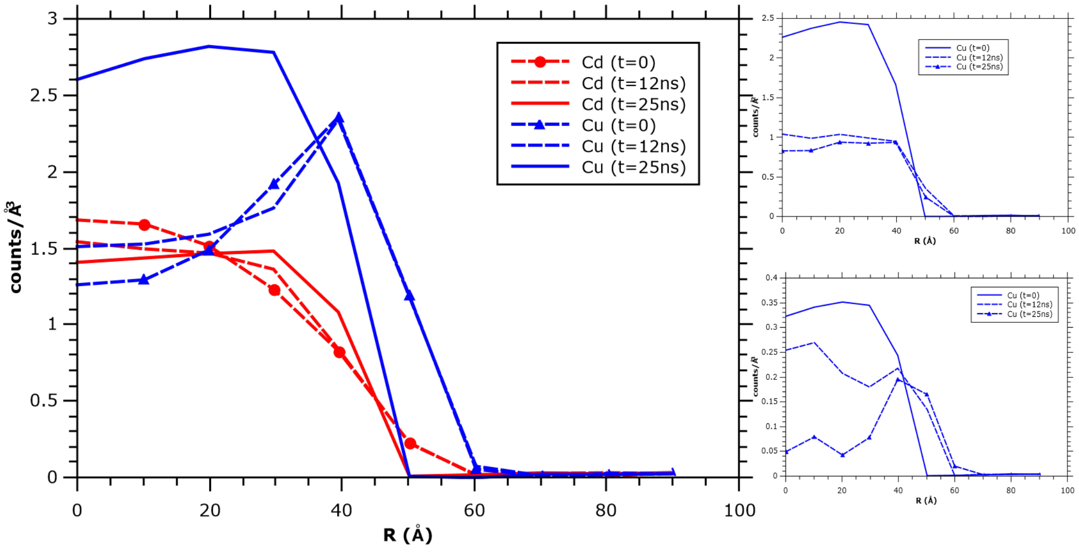

Figure SI_11. MS3 (left panel): starting and following evolution of Cu (blue atoms) and Cd (red atoms) radial distribution at diverse times (0, 12 and 25 ns). Right top panel: Cu radial evolution in the portion of MS3 containing only the larger Cu_2Se domain between two thin layers of zb CdSe. Right bottom panel: Cu radial evolution in the portion of MS3 containing only the smaller Cu_2Se domain with a thin layer of zb CdSe on its top.

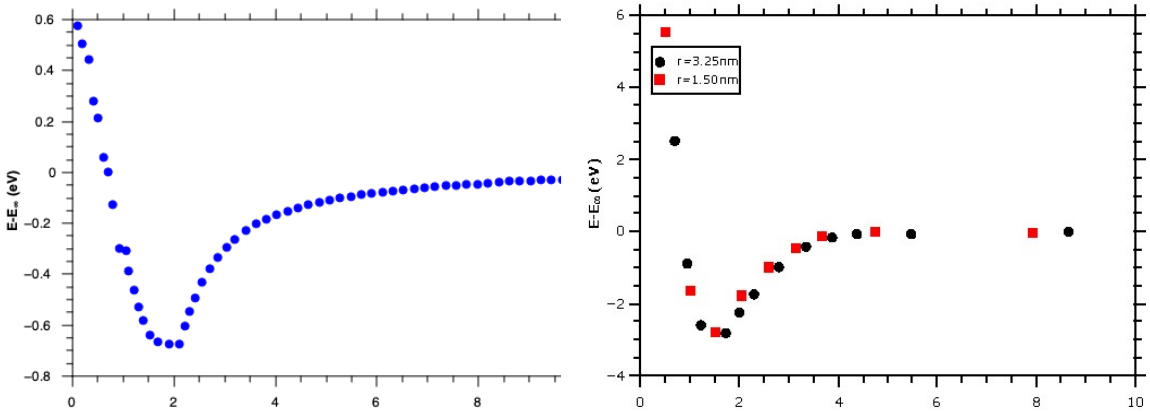

Figure SI_12. Attraction basin for a Cu atom close to a CdSe NW. Left panel corresponds to the new FF and the right panel to DFT.

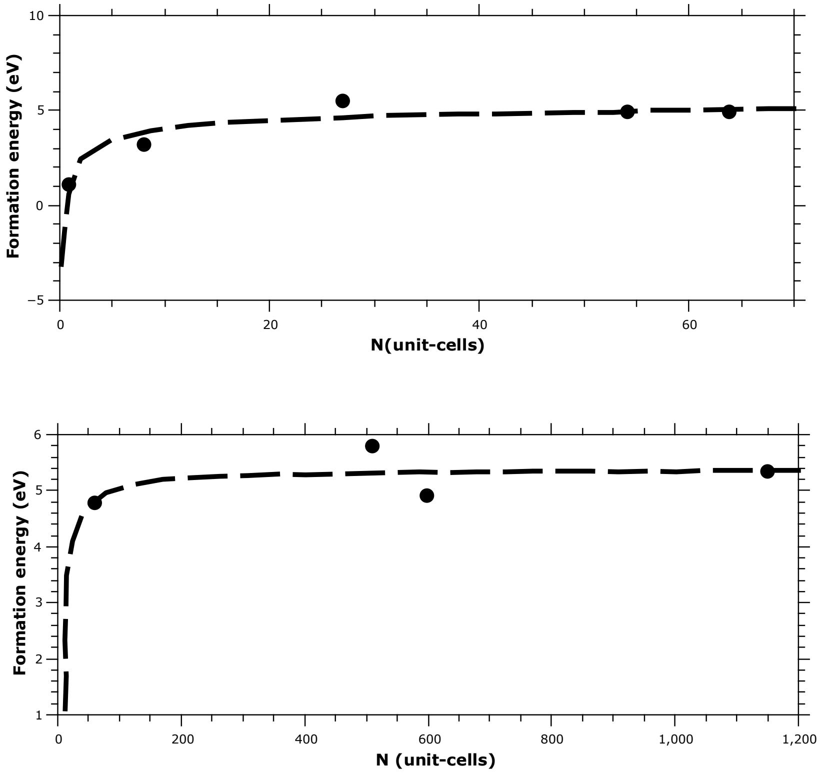

Figure SI_13. Cd vacancy formation energy as a function of the computational cell size estimated using DFT (top) and our FF (bottom).

Video_S1: The animated video titled "Video_S1" summarizes what happens to a zincblende CdSe NW and a population of copper atoms thermally expelled by Cu_2Se NCs when the temperature increases up to 125 °C (about 400 K). At first (*i.e.*, at temperature lower than 125 °C), copper atoms start constituting a very thin and homogenous layer around the NWs. Once the T=125 °C is reached, the Cu atoms enter the CdSe NWs to kick-off and replace the Cd cations. Thus, at the end of the whole process, the original CdSe NW with zb structure becomes a Cu_2Se NW.
